# Factors associated with longer brain metastasis-free survival in limited-disease small-cell lung cancer who underwent concurrent chemoradiotherapy, a retrospective analysis

**DOI:** 10.3389/fonc.2025.1739788

**Published:** 2026-01-19

**Authors:** Xiang-Rong Zhao, Jia Li, Zhen Zhang, Chao Gao, Zheng-Qiang Yang

**Affiliations:** 1 Department of Radiation Oncology, Liao Cheng People's Hospital, Liao Cheng, Shandong, China; 2 Department of Radiation Oncology, Shandong Cancer Hospital and Institute, Shandong First Medical University and Shandong Academy of Medical Sciences, Jinan, Shandong, China; 3 Department of Radiation Oncology, The Third Affiliated Hospital of Shandong First Medical University, Jinan, Shandong, China

**Keywords:** brain metastases, chemoradiotherapy, PCI, risk factors, small cell lung cancer

## Abstract

**Introduction:**

This study aimed to explore the limited-disease small-cell lung cancer (LD-SCLC) patients who were treated with concurrent chemo-radiotherapy (CCRT) without prophylactic cranial irradiation (PCI), and evaluated the possible risk factors for BM development in this population.

**Methods:**

This study was a retrospective study which reviewed 182 patients treated with CCRT for stages I-III and who finally developed brain metastases (BM) between 2017 and 2021 were reviewed to elucidate the risk factors for BM.

**Results:**

In this study, N descriptors at initial SCLC (N3, 5.9 points; N2, 3.1 points; N1, 1.5) and response of primary tumor (PT) to CCRT (NR 4.7; PR 1.0) were most strongly associated with BM-free survival. Based on these factors, patients were stratified as low risk (4.0 or fewer total points), moderate risk (4.1 to 8.0 points), and high-risk (more than 8.0 points), and the median BM-free survival was 24 months, 16 months, and 8 months, respectively, with 12-month BM-free survival of 91.7%, 66.3%, and 25.0%, respectively (*P* <0.001).

**Discussion:**

Our results demonstrate that N descriptors and remission grade of the primary tumor after CCRT were significantly correlated with the incidence of BM.

## 1 Introduction

Small cell lung cancer (SCLC) is characterized by rapid tumor growth, early dissemination, a high incidence of metastatic disease at presentation, and poor prognosis (1). Autopsy studies have confirmed that the brain is a major sanctuary site in SCLC (2). Brain metastases (BM) is common in patients with SCLC; approximately 15%-20% of SCLC patients have detectable brain metastases at the time of initial diagnosis, and the incidence of BM increases as high as 50%-65% at postmortem examination (3, 4). To our knowledge, maintenance chemotherapy has not demonstrated the ability to reduce the incidence of subsequent metastases after primary therapy (5–7). Moreover, BM is associated with poor prognosis, and the median survival after BM development is only 4-6 months (3, 8, 9).

In the 1990s, a number of randomized trials unequivocally showed that prophylactic cranial irradiation (PCI) reduced the incidence of BM in patients with limited-disease small cell lung cancer(LD-SCLC), without increasing toxicity if not administered concurrently with chemotherapy (10–12). In contrast, Fleck et al. was not supporting the use of PCI given the lower efficacy and significant increase in their toxicity (13). Other retrospective studies in the 1980s also raised concerns about the potential neuropsychological toxicity of PCI, such as ataxia, dementia, and cognitive decline (14). Previous studies have emphasized the significance of factors such as tumor size and treatment response in predicting BM (15). Larger tumors and partial responses to treatment are associated with an increased risk of BM. Research into molecular and genetic markers, such as circulating tumor cells (CTCs) and specific gene mutations, holds promise for more accurately predicting BM risk (16). Integrating these biomarkers into clinical practice could lead to more personalized treatment approaches, thereby improving survival rates and quality of life for LD-SCLC patients (17).

We reviewed patients with LD-SCLC treated with concurrent chemo-radiotherapy (CCRT) without PCI, and evaluated the possible factors associated with earlier BM occurrence in this population.

## 2 Methods

### 2.1 Patients

We reviewed the medical records of all adult patients who were diagnosed with histologically proven SCLC between July 2017 and October 2021 at Shandong Cancer Hospital and Institute. Exclusion criteria included incomplete chart information. Patients with other concurrent malignancies or combined-type SCLC (SCLC with squamous cell carcinoma and/or adenocarcinoma) were excluded from this study. Data were collected retrospectively, including physical examination, treatment, chest radiography, contrast-enhanced computed tomography (CT) scans of the chest and upper abdomen, magnetic resonance imaging of the brain, bone radionuclide imaging, and cyto-histopathological results of transthoracic needle biopsy or bronchoscopic biopsy. All patients had no brain metastases on MRI and PCI was not performed. All study patients who had been classified using the two-stage system were restaged according to the UICC for International Cancer Control 7th TNM staging. The current study was performed in accordance with the Declaration of Helsinki and approved by the local ethics committee of the Shandong Tumor Hospital (Jinan, China; approval no. FY2017015). Each patient provided written informed consent for participation.

All patients signed written informed consent for inclusion of their clinical details in the manuscript for publication.

### 2.2 Treatment

All patients received either cisplatin or etoposide (EP: 30 mg/m^2^ cisplatin on days 1-3 and either 100 mg/m^2^ etoposide on days 1-5 or 100 mg/m^2^ etoposide on days 1-3) or carboplatin and etoposide (CE: carboplatin AUC 5 or 300 mg/m^2^ on day 1 and either 100 mg/m^2^ etoposide on days 1-5 or etoposide 100 mg/m^2^ on days 1-3). The median number of cycles of chemotherapy was 5.

Thoracic radiation therapy (TRT) was administered using intensity modulated radiation therapy technique. The target volume for thoracic radiotherapy included the gross tumor, as defined by the chest CT scan, and the bilateral mediastinal and ipsilateral hilar lymph nodes. This has been our institutional practice though we acknowledge many other centers internationally have moved to omission of elective nodal irradiation and primarily target PET-positive disease with margin. The inferior border extended 3 cm below the carina or to a level including the ipsilateral hilar structures, whichever was lower. The clinical target volume (CTV) was expanded by a margin of 1 to 1.5 cm, and the planning target volume (PTV) included the CTV with a 0.5 cm margin. The radiation was delivered using megavoltage linear accelerators.

TRT was initiated within one week of the second cycle of chemotherapy. Patients receiving once-daily therapy received 1.8-2.0 Gy/day to 42-66 Gy over a period of five weeks. Accelerated twice-daily thoracic radiotherapy involved administration of 1.5 Gy over a period of three weeks.

### 2.3 Follow-up

The follow-up schedule started from the time of the first treatment. Long-term outcomes were obtained from the hospital records or correspondence with patients, families. The last follow-up was conducted on March 31, 2023. CT scans were obtained after the second and fourth cycles of chemotherapy for tumor assessment and every 8-12 weeks thereafter for further follow-up. Response to therapy was independently re-evaluated within 2 weeks after CCRT completion and based on CT scanning, in accordance with the Response Evaluation Criteria in Solid Tumors (RECIST, version. 1.1) (18).

### 2.4 End points

The primary endpoint in this study was BM-free survival time, defined as the period from the initial diagnosis to the date of cerebral metastasis, in patients whose first site of recurrence was the brain.

### 2.5 Statistical analysis

Descriptive statistics for clinical variables are presented as median and interquartile range (IQR), or as numbers with percentages, as appropriate. Descriptive statistics for BM-free survival time are shown as Hazard ratio (HR) and 95% confidence intervals (95% CI). The actuarial risk of developing BM and BM-free survival time curves were estimated using the Kaplan-Meier method. The log-rank test was used to compare the differences between the groups. Cox proportional hazards regression models were used to develop univariate and multivariate models describing the association between independent variables and BM-free survival time. All reported characteristics were considered in a multivariable Cox model to determine which demonstrated the strongest association with the BM-free survival time. A forward stepwise selection procedure was implemented with a two-sided *p*-values threshold of ≤ 0.05 for inclusion in the final model. The HR and 95% CI were reported for the variables included in the final model. *P*-values of ≤0.05 were considered statistically significant. All analyses were performed using SPSS version 22.0 software.

The final multivariable model was converted to a risk score for BM using the following steps: (1) the variable with the smallest regression coefficient in the model was assigned 1.0 risk score points; (2) risk score points were assigned to all other variables in the model by dividing their respective regression coefficients by the smallest coefficient in the model; this quotient rounded to the nearest tenth is the risk score point assignment for that variable; and (3) a total risk score was calculated for every patient by adding the risk score points assigned to each variable in the model (19). The set of total risk scores was divided into three groups, and the Kaplan-Meier survival curves for each juxtaposed to qualitatively assess the risk score’s ability to discriminate between patients with high and low risk of BM. The discriminating ability of the risk score model was measured quantitatively by the c-statistic.

## 3 Results

### 3.1 Patients characteristics

In total, the records of one hundred and eighty-two patients were enrolled and analyzed in this study, among which 182 patients (117 men and 65 women, all Han Chinese) completed the described treatment and developed BM. The patient characteristics are summarized in **Table 1**. The median follow-up period for all patients was 57 months (range, 30-81 months). The median age at diagnosis was 57 years (range, 32-80 years).

**Table 1:** Characteristics of patients (n = 182).

Characteristics	N	%
Number of patients	182	
Age		
Median (years)	57	
Range (years)	32–79	
Sex		
Male	117	64.3%
Female	65	35.7%
Age		
>57	91	50.0%
≤57	91	50.0%
ECOG PS		
0,1	137	75.3%
≥2	45	24.7%
Weight loss		
≥5%	158	86.8%
<5%	24	13.2%
Smoking status		
Yes	52	28.6%
no	130	71.4%
Complication		
Yes	38	20.9%
no	144	79.1%
Bone marrow suppression		
≤1	72	39.5%
≥2	110	60.5%
Gastrointestinal reaction		
≤1	139	76.4%
≥2	43	23.6%
Number of cycles		
<4	5	2.7%
4–6	150	82.4%
>6	27	4.9%
RT dose		
≥56	100	54.9%
<56	82	45.1%
TRT fractions		
Once–daily	171	93.9%
Twice–daily	11	6.1%
Response of PT to CRT		
CR	87	47.8%
PR	49	26.9%
NR	46	25.3%
T descriptor		
T1	38	20.9%
T2	56	30.8%
T3	38	20.9%
T4	50	27.4%
N descriptor		
N0	5	2.7%
N1	18	10.0%
N2	49	26.9%
N3	110	60.4%
TNM Stage Grouping		
I	4	2.2%
II	24	13.2%
III	154	84.6%

ECOG PS, Eastern Cooperative Oncology Group Performance Status, RT, radiotherapy, PT, primary tumor, CRT, chemoradiotherapy, CR, complete response, PR, partial response, NR, no response.

### 3.2 Descriptive of BM-free survival time

**Figure 1:**
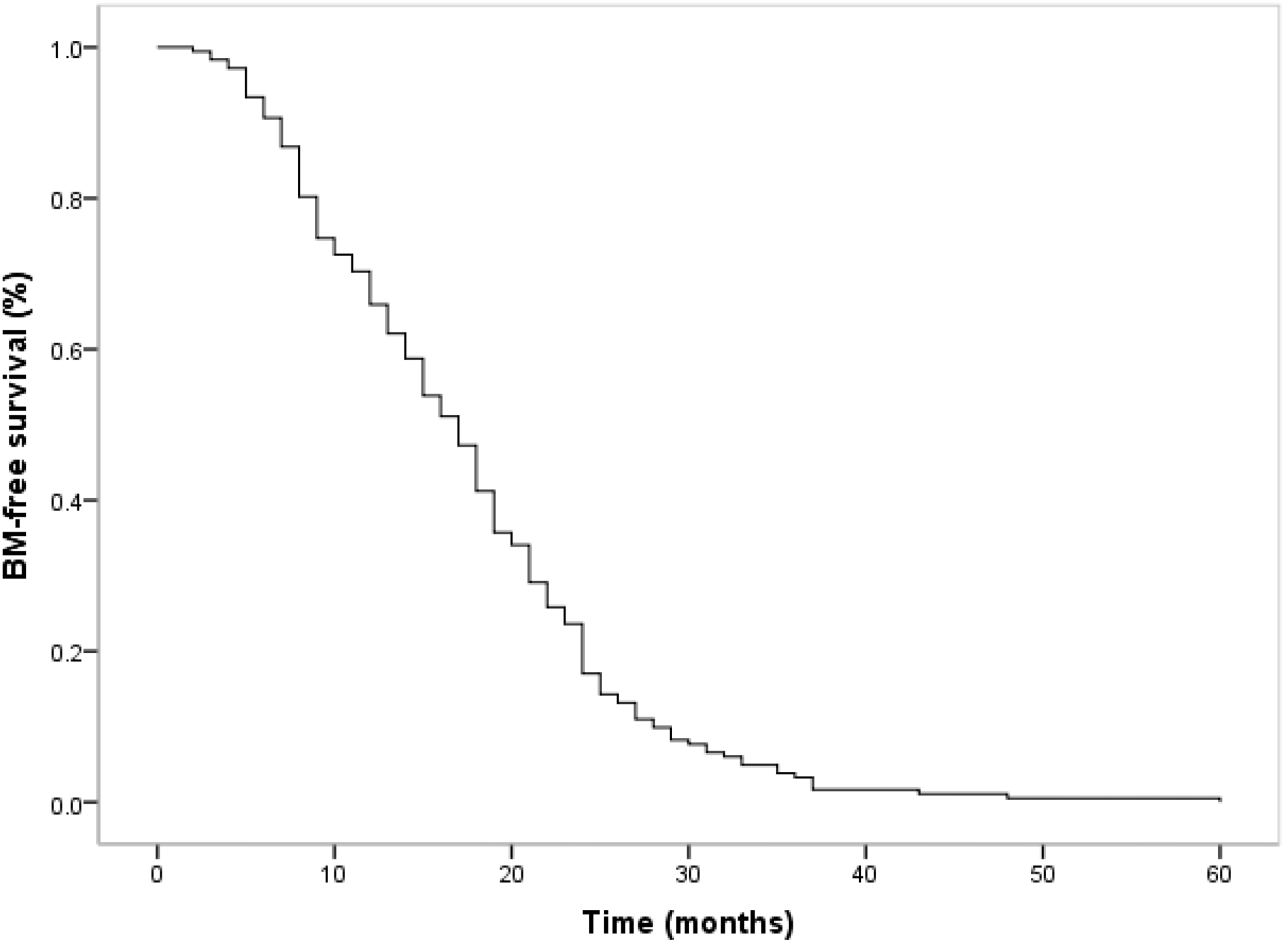
BM–free survival time curve for 182 patients in small cell lung cancer with concurrent chemoradiotherapy.

All our patients finally developed BM. The time for the development of BM ranged from 2 months to 60 months, which is a natural process after systemic treatment, with a median time from initial diagnosis to BM development 17 months (25^th^ and 75th percentiles: 9, 23). IQR = 75^th^ percentile – 25^th^ percentile: 14 months. The median ± IQR for the BM-free survival time was 17 ± 14 months. The estimated BM-free survival rates at 12 and 24 months were 65.9% and 17.0, respectively (**Figure 1**).

### 3.3 Univariate BM-free survival analyses

**Figure 2:**
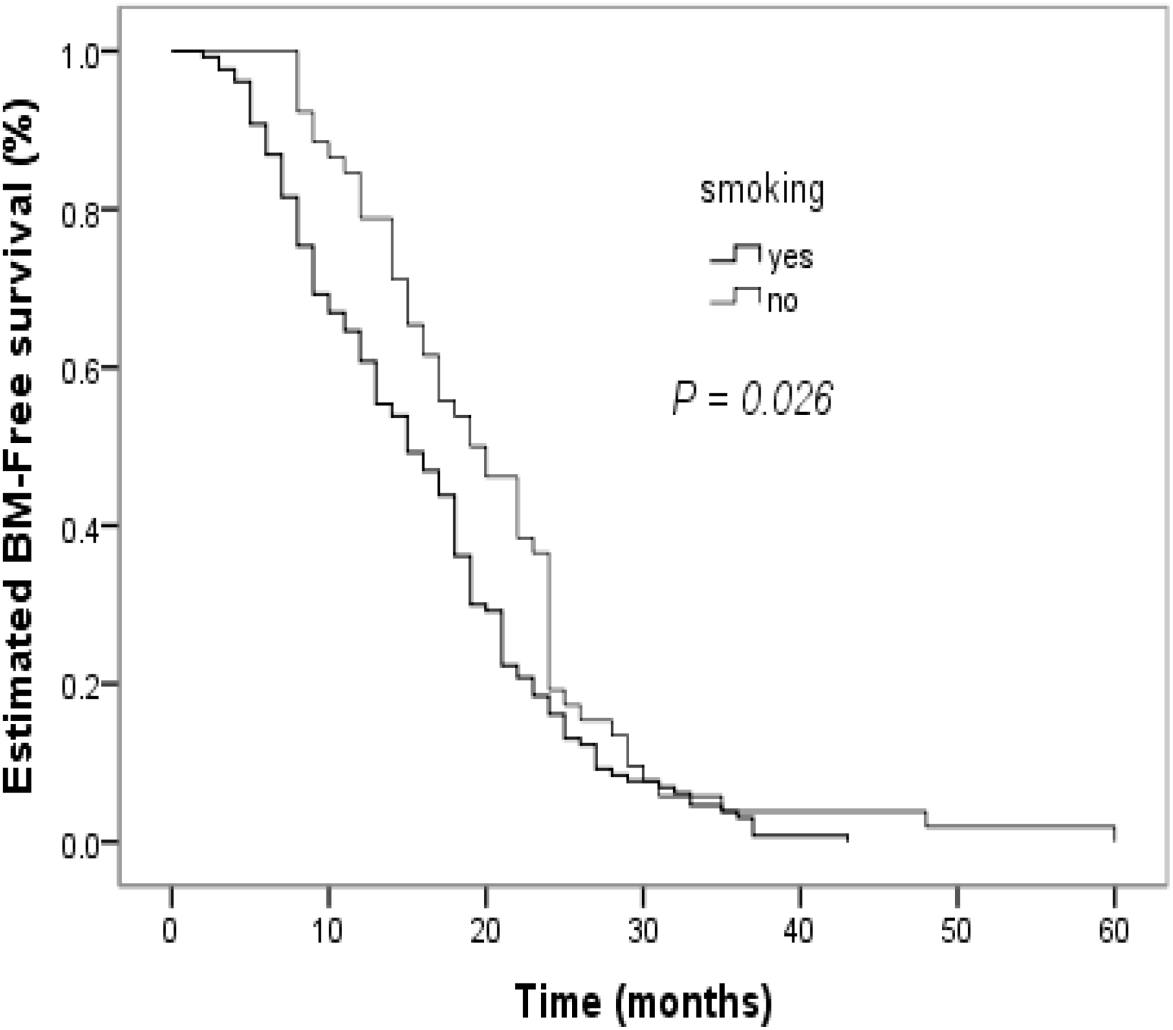
Comparison of BM–free survival among patients with small cell lung cancer based on smoking in Kaplan–Meier analyses, which indicated that smoking status influenced BM–free survival time in non–smoking versus smoking (*P* = 0.026).

**Figure 3:**
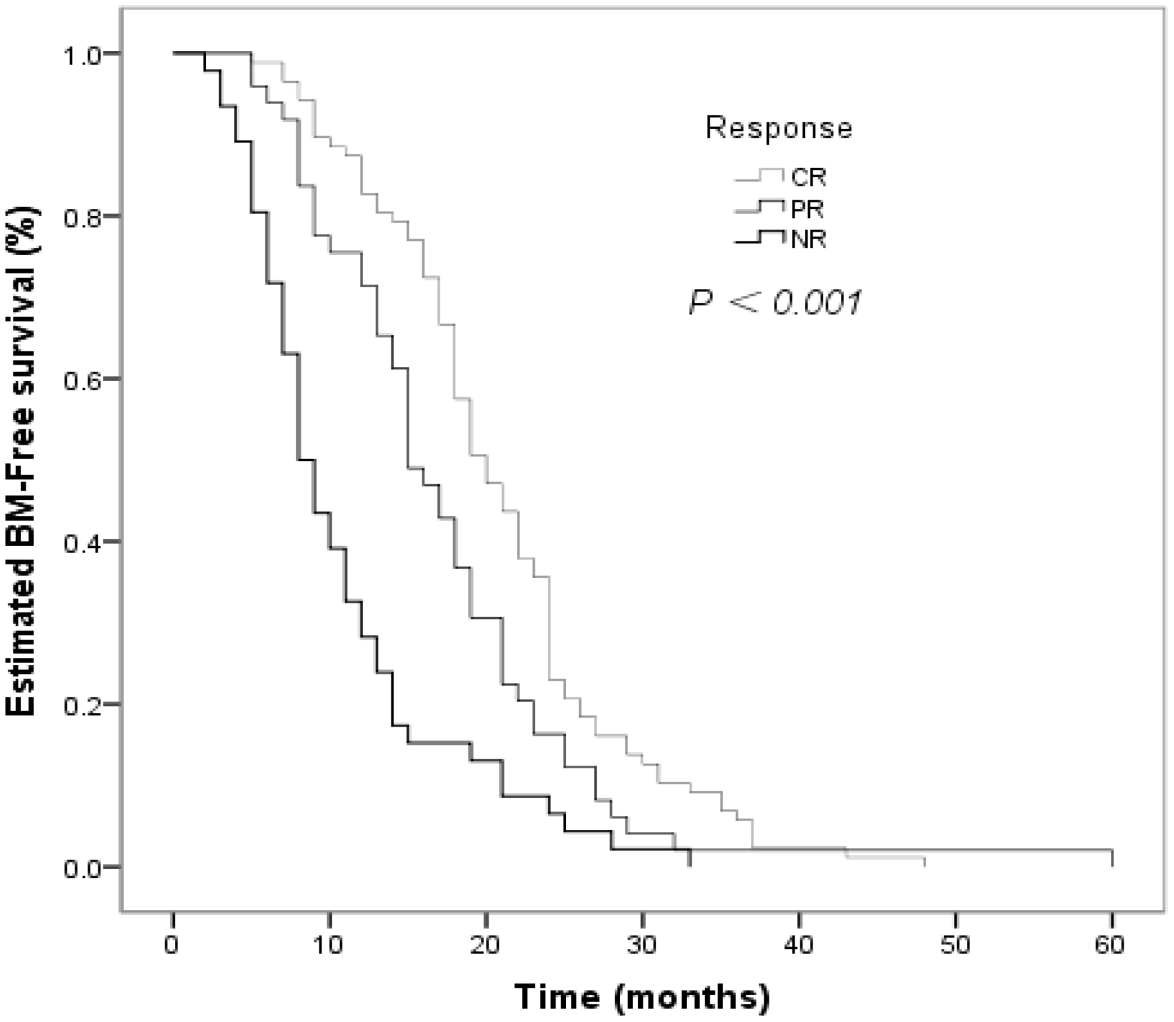
Comparison of BM–free survival among patients with small cell lung cancer based on response of primary tumor (PT) to concurrent chemoradiotherapy (CCRT) in Kaplan–Meier analyses, which indicated that there was significant difference in BM–free survival time according to the response of PT to CCRT (*P*  < 0.001).

**Figure 4:**
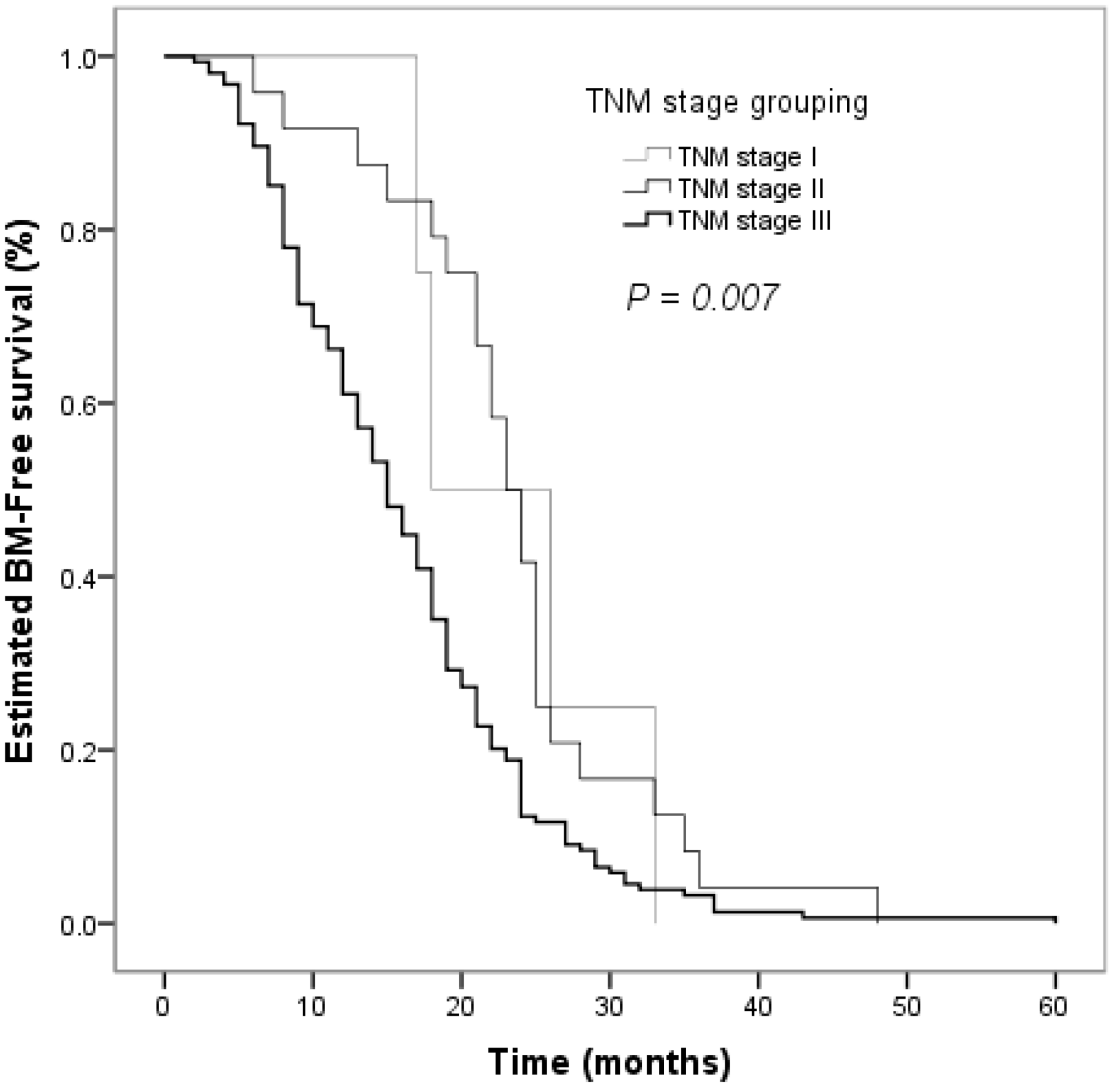
Comparison of BM–free survival among patients with small cell lung cancer based on TNM stage grouping in Kaplan–Meier analyses, which indicated that TNM stage groupings of the initial SCLC were strongly associated with BM–free survival time (*P* = 0.007).

**Figure 5:**
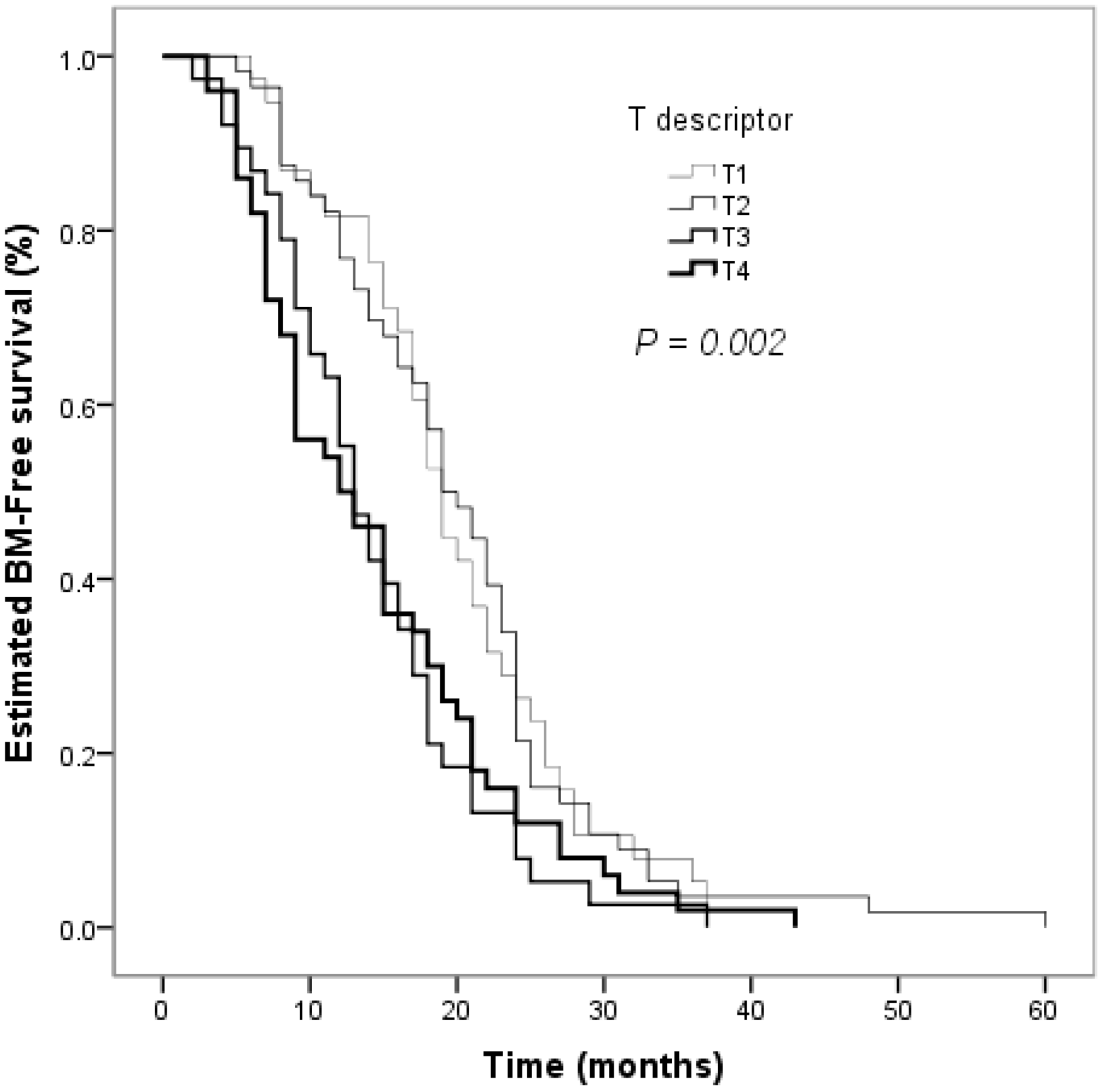
Comparison of BM–free survival among patients with small cell lung cancer based on T descriptors in Kaplan–Meier analyses, which indicated that the risk of developing BM was associated with T descriptors (*P* = 0.002).

**Figure 6:**
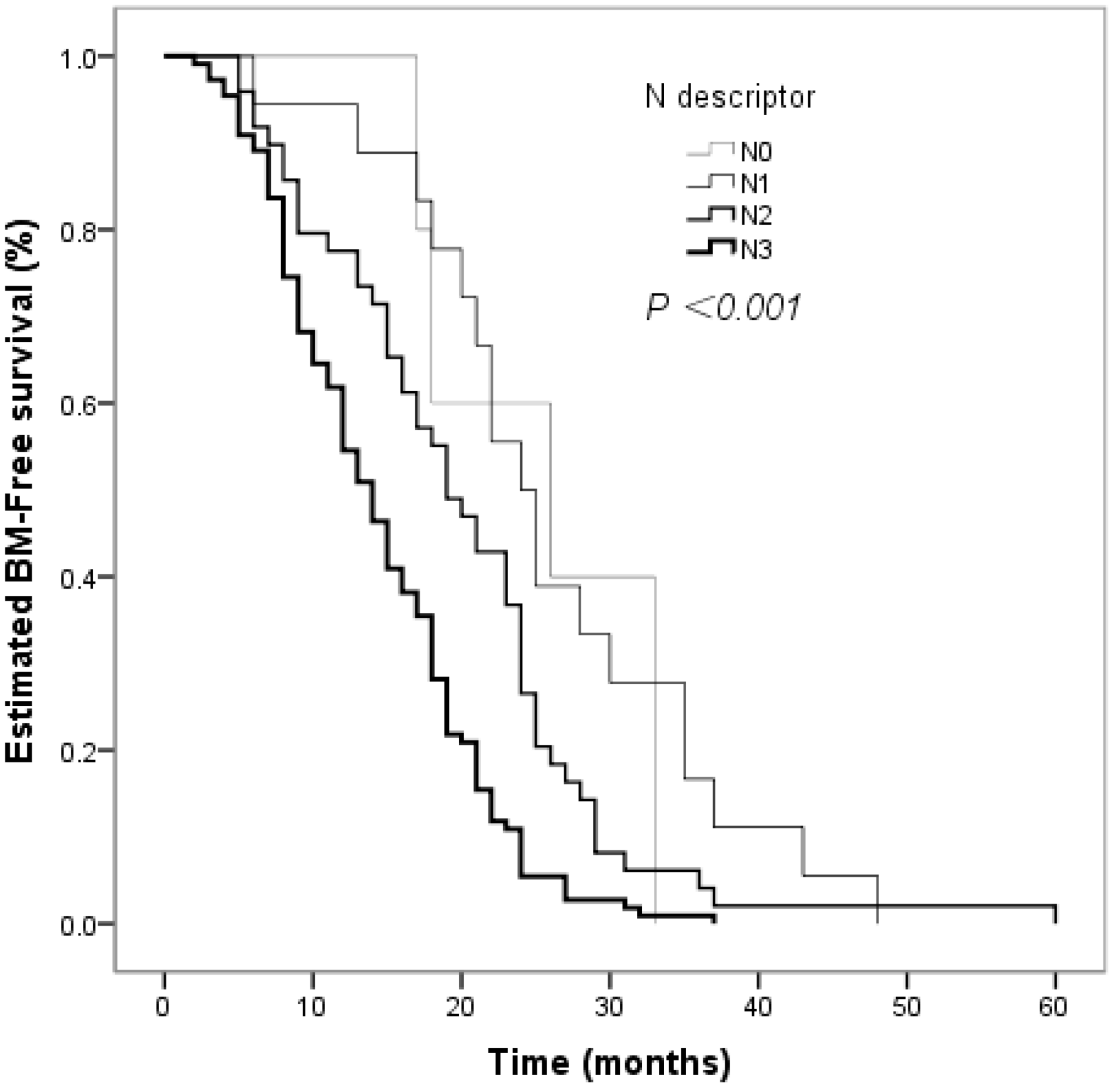
Comparison of BM–free survival among patients with small cell lung cancer based on N descriptors in Kaplan–Meier analyses, which indicated that N descriptors were also associated with BM–free survival time (*P* < 0.001).

The clinical factors evaluated in the univariate analyses to determine their prognostic value for the risk of developing BM are shown in **Table 2**. Smoking status influenced the BM-free survival time in non-smokers and smokers (*P* = 0.026, **Figure 2**). The estimated 12-month and 24-month BM-free survival times were 60.8% and 16.2% in patients who smoked; and 78.8% and 19.2% in patients who did not smoke. There was a significant difference in the BM-free survival time according to the response of PT to CCRT (*P*  < 0.001, **Figure 3**). The estimated 12-month and 24-month BM-free survival times were 82.8% and 23.0 in patients with CR; 71.4% and 16.3% in patients with PR; and 28.3% and 6.5% in patients with NR. TNM stage grouping of the initial SCLC showed detrimental effects on the BM-free survival time (*P* = 0.007, **Figure 4**). The estimated 12-month and 24-month BM-free survival rates were 87.5% and 41.7% in patients with stage II disease and 61.0% and 12.3% in patients with stage III disease. The risk of developing BM was associated with the T descriptors (*P* = 0.002, **Figure 5**). The estimated 12-month and 24-month BM-free survival times were 81.6% and 26.3%, 76.8% and 21.4%, 55.3% and 7.9%, 50.0% and 12.0%, in patients with T1 disease T2 disease, T3, and T4 disease, respectively. N descriptors were also associated with the BM-free survival time (*P* < 0.001, **Figure 6**). The estimated 12-month and 24-month BM-free survival times were 88.9% and 50.0%, 77.6 and 26.5, 54.5% and 5.5% in patients with N1, N2, and N3 disease, respectively. We did not describe patients with stage I or N0 disease because only four patients and five patients respectively.

**Table 2:** Associations between small cell lung cancer characteristics and brain metastases in univariate analysis.

	N	%	HR(95%CI)^a^	*P* Value
Sex Male	117	64.3	0.98 (0.72, 1.32)	0.88
Age ≥57	91	50	0.93 (0.69, 1.24)	0.61
ECOG PS ≥2	45	24.7	0.96 (0.68, 1.34)	0.79
Weight loss ≥5%	24	13.2	0.71 (0.46, 1.10)	0.13
Complication Yes	38	20.9	1.28 (0.89, 1.83)	0.18
Gastrointestinal reaction ≥2	43	23.6	1.13 (0.80, 1.60)	0.50
Bone marrow suppression ≥2	110	60.4	1.08 (0.80, 1.44)	0.67
TRT fractions once–daily	171	94.0	0.88 (0.48, 1.62)	0.68
RT Dose ≥56	100	54.9	0.78 (0.58, 1.06)	0.11
CHT Cycle				0.09
>6	27	14.8	0.42 (0.16, 1.10)	
4–6	150	82.4	0.63 (0.26, 1.54)	
<4	5	2.8	1.00(–)	
No Smoking	130	71.4	0.70 (0.51, 0.98)	0.035
Response				< 0.001
NR	46	25.3	3.11 (2.15, 4.49)	
PR	49	26.9	1.45 (1.01, 2.06)	
CR	87	47.8	1.00 (–)	
T descriptor				0.004
T4	50	27.5	1.61 (1.05, 2.45)	
T3	38	20.9	1.76 (1.12, 2.77)	
T2	56	30.7	0.95 (0.63, 1.45)	
T1	38	20.9	1.00 (–)	
N descriptor				< 0.001
N3	110	60.4	2.91 (1.17, 7.21)	
N2	49	27.0	1.50 (0.59, 3.79)	
N1	18	9.9	0.90 (0.33, 2.45)	
N0	5	2.7	1.00 (–)	
TNM stage grouping				0.012
III	154	84.6	1.80 (0.66, 4.86)	
II	24	13.2	0.96 (0.33, 2.78)	
I	4	2.2	1.00 (–)	

^a^Relative risk (95% confidence interval), ECOG PS, Eastern Cooperative Oncology Group Performance Status, RT, radiotherapy, CRT, chemoradiotherapy, CR, complete response, PR, partial response, NR, no response.

### 3.4 Multivariate Cox proportional hazards regression analyses

Multivariate Cox proportional hazards regression analysis showed that PT response to CCRT (HR = 1.609, 95%CI: 1.324-1.954, *P* < 0.001) and N descriptor (HR = 1.564, 95%CI: 1.268-1.929, *P* < 0.001) were independent factors associated with an increased risk of developing BM (**Table 3**). The final multivariable model of the risk factors for BM-free survival time with the corresponding risk score points is shown in **Table 3**. This model included two characteristics related to BM-free survival (N descriptors and PT response to CCRT). Risk score points were assigned as shown for each of the two listed factors, with a total risk score calculated per patient as the sum of the risk score points for all two factors. **Table 4** shows the risk score calculations for the 7 most commonly observed combinations of factors in our patient population. For instance, as shown in the third row of **Table 4**, an individual with stage N3 disease (5.9 risk score points), and complete response of PT to CCRT (0.0 risk score points) would be assigned a risk score of 5.9 points (risk score = 5.9 + 0.0 = 5.9).

**Table 3:** Multivariate analyses of risk factors and risk score (RS) points assignments for BM–free survival in patients with small cell lung cancer.

Characteristic	HR^a^ (95% CI)	p Value	RS^b^ Points
N descriptor		< 0.001	
N3	3.65 (1.45, 9.24)		5.9
N2	1.99 (0.77, 5.12)		3.1
N1	1.38 (0.49, 3.85)		1.5
N0	1.00 (ref)		0.0
Response		< 0.001	
NR	2.83 (1.93, 4.15)		4.7
PR	1.25 (0.87, 1.79)		1.0
CR	1.00 (ref)		0.0

^a^Hazard ratio (95% confidence interval). ^b^Risk score.

BM, brain metastases, ref, reference, CR, complete response, PR, partial response, NR, no response.

**Table 4:** Seven most commonly observed combinations of risk factors among patients with BM.

Number of patients	N descriptors at initial SCLC	Response of PT to CRT	Risk score^a^
14(8.1%)	N1 (1.5)^b^	CR (0.0)	1.5
26(15.1%)	N2 (3.1)	CR (0.0)	3.1
42(24.4%)	N3 (5.9)	CR (0.0)	5.9
13(7.6%)	N2 (3.1)	PR (1.0)	4.1
33(19.2%)	N3 (5.9)	PR (1.0)	6.9
9(5.3%)	N2 (3.1)	NR (4.7)	7.8
35(20.3%)	N3 (5.9)	NR (4.7)	10.6

^a^Total risk score calculated as the sum of risk score points for the two factors. ^b^Risk score points are given in parentheses.

BM, brain metastases, SCLC, small cell lung cancer, PT, primary tumor, CRT, chemoradiotherapy, CR, complete response, PR, partial response, NR, no response.

### 3.5 Measurement of risk score groups

**Figure 7:**
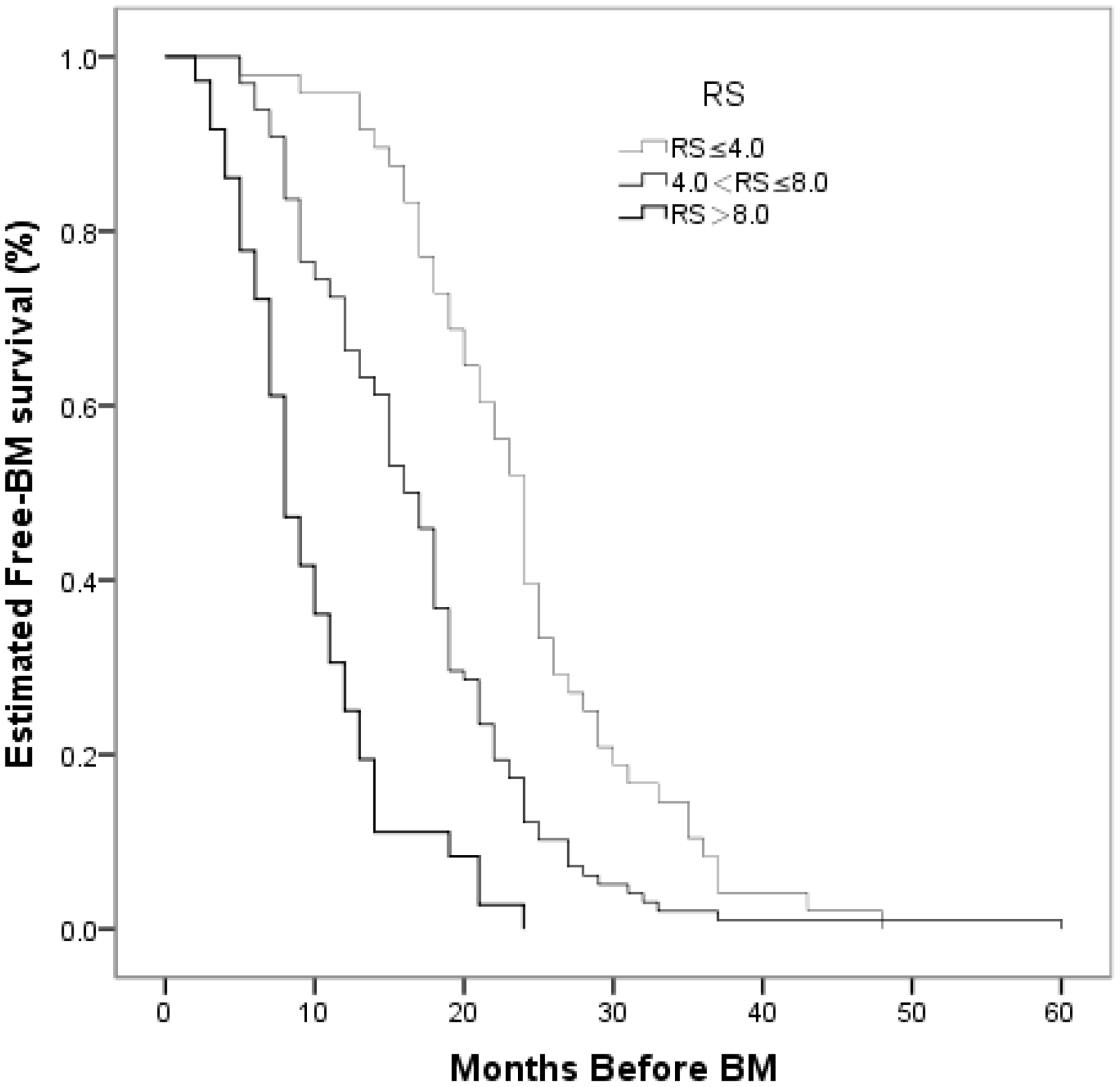
Comparison of BM–free survival among patients with small cell lung cancer based on risk score in Kaplan–Meier analyses. The difference was statistically significant among the three groups. (*P* < 0.001).

**Figure 8:**
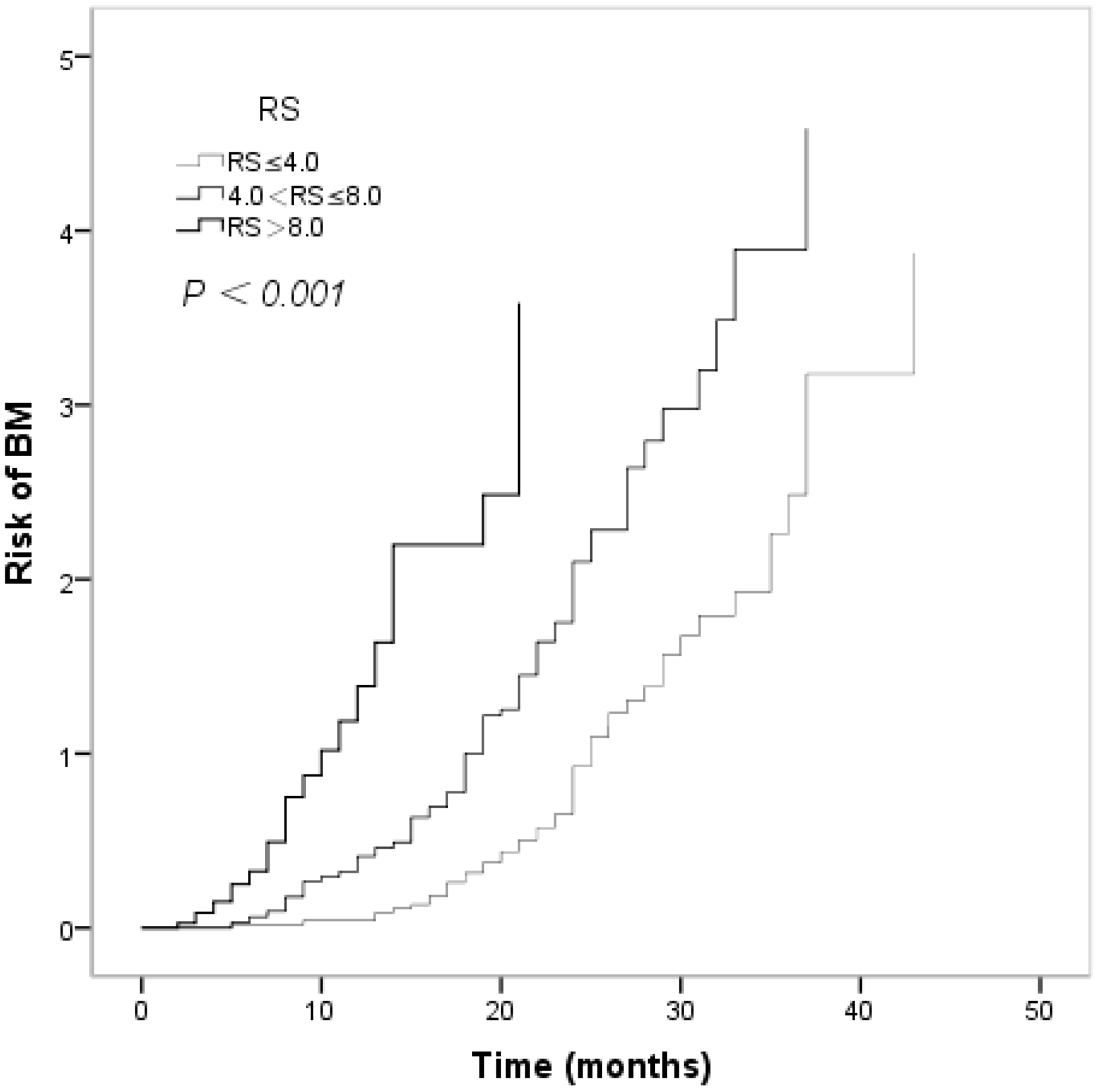
The acturatial risk of developing brain metastasis in patients with different risk score of small cell lung cancer.

Risk score groups were created empirically and referring to Brent^’^s study (20) as follows: (1) risk score of 4.0 or less (low risk); (2) risk score of 4.1 to 8.0 (moderate risk); and (3) risk score greater than 8.0 (high risk). In univariate analyses, the differences were statistically significant among the three groups. The estimated 12-month BM-free survival rates across groups were 91.7%, 66.3%, and 25.0%, respectively. The estimated 24-month BM-free survival rates across the groups were 39.6%, 12.2%, and 0.0%, respectively (*P* < 0.001, **Figure 7**). The actuarial risk of BM development in the three groups is shown in **Figure 8**.

## 4 Discussion

Smoking is the main risk factor for SCLC; more than 90% of patients with SCLC are elderly or past heavy smokers, and the risk increases with increasing duration and intensity of smoking (21). Earlier studies have documented that the brain is a prevalent site of failure in LD-SCLC and suggested that the development of BM is an indicator of very poor prognosis (22–24). Additionally, Videtic et al. reported that patients with SCLC who smoked had increased toxicity during treatment and shorter survival (25). However, some authors did not find a relationship between BM–free survival time and smoking (9, 26). Our results are consistent with those of previous studies. On the other hand, our patients who smoked had significantly lower BM–free survival times in the univariate analysis. These discrepancies may be due to differences in treatment.

Recently, the International Association for the Study of Lung Cancer (IASLC) proposed that the newly revised tumor, node, metastasis (TNM) staging system should be applied to SCLC because the T and N descriptors, as well as the overall stage I to IV groupings, are discriminatory for survival (27). Our data for the T descriptor showed a significant correlation between T descriptors and BM–free survival in univariate analysis. Similar observations were reported as follows: Ignatius and colleagues revealed that the current UICC6 and IASLC proposed T descriptors were correlated with survival (28). Frances et al. were also consistent with the above analysis (27). However, there have been studies with results that are not similar to ours. For example, Lim et al. evaluated survival in 59 patients with LD–SCLC who underwent surgical resection or adjuvant chemotherapy and found no clear survival difference according to T descriptors (29). The contradictory findings for the T descriptors may be partly explained by the characteristics of the tumors, which usually present with bulky lymphadenopathy and without a lung tumor or conglomerated lesion (30, 31), making the accurate assessment of T descriptors difficult.

In our study, our results for N descriptors also showed a significant correlation between N descriptors and BM–free survival in univariate and multivariate analyses and were identified as independent risk factors for BM–free survival. This finding is similar to the results of previous studies. Angeletti et al. evaluated the influence of T and N stages on the long–term survival of 49 patients with SCLC who underwent surgical resection or adjuvant chemotherapy. They showed that survival was significantly influenced by N, but not by T descriptors (30). Ichinose et al. investigated the correlation between the development of BM and pre–treatment TNM stage in patients with LD–SCLC who achieved complete remission by CRT or curative surgery and found that the incidence of subsequent BM was significantly higher in patients with advanced tumor stage and nodal status (32).

Ichinose et al. also demonstrated that the incidence of BM after treatment was correlated with the pretreatment clinical stage. In patients with stage I and II SCLC, the incidence was 17.4% (4/23), whereas it was 59.1% (12/22) in patients with stage III SCLC (*P* < 0.005) (29). Additionally, Nakamura et al. observed that BM occurred in 7% (2/30) of patients with stage I disease, 25% (3/12) of patients with stage II disease, and 27% (7/26) of patients with stage III disease (33), which revealed that TNM stages I–III were significantly associated with the incidence of BM. Our study demonstrated a significant correlation between TNM stages I–III and BM–free survival in univariate analysis, which was similar to the results reported by others.

In a previous study, Manapov and colleagues reported that the response of primary tumors to CRT strongly correlates with the duration of BM–free survival and rate of subsequent development of BM, and that the duration of BM–free survival was significantly shorter in the poor– and partial responders compared to complete responders and was independent of the type of applied multimodality treatment, which suggested that achievement of complete response of the primary tumor to CRT positively influences the course of SCLC disease due to significant prolongation of BM–free survival time (34).

We observed a strong association between the response of the primary tumor to CCRT and the duration of BM–free survival in univariate and multivariate analyses and identified them as independent risk factors for BM–free survival, which is in good accordance with the above report and some previous clinical trials demonstrating that by improvement of the local, distant control can be emended (35, 36).

The primary limitation of this study was that data loss could have occurred because of the retrospective design. The number of patients in stage **Ⅰ–Ⅱ** was small. All patients eventually developed brain metastases, so this study has only included those with a poorer prognosis. The study cannot estimate incidence or risk of developing BM, only time to BM. So this study only evaluated the possible factors associated with earlier BM occurrence in this population. Immunotherapy trials have provided limited data on its impact on patients who treated with PCI. The IMpower 133 (37) and CASPIAN trials (38) suggested that adding immunotherapy could delay intracranial relapse. In the immunotherapy era, the therapeutic status of PCI still faces important challenges. Further prospective, randomized, controlled studies are needed to confirm these findings with a greater power effect.

## 5 Conclusions

We found that the response of PT to CCRT and N descriptors were independent risk factors for the duration of BM–free survival in LD–SCLC patients. The study suggests that the remission grade of the primary tumor and advanced N descriptors influence the course of LD–SCLC and should be considered when planning sufficient treatment.

## Data Availability

The original contributions presented in the study are included in the article/supplementary material. Further inquiries can be directed to the corresponding author.
