# Correction: Factors associated with longer brain metastasis-free survival in limited-disease small-cell lung cancer who underwent concurrent chemoradiotherapy, a retrospective analysis

**DOI:** 10.3389/fonc.2026.1791673

**Published:** 2026-01-27

**Authors:** Xiang-Rong Zhao, Jia Li, Zhen Zhang, Chao Gao, Zheng-Qiang Yang

**Affiliations:** 1 Department of Radiation Oncology, Liao Cheng People’s Hospital, Liao Cheng, Shandong, China; 2 Department of Radiation Oncology, Shandong Cancer Hospital and Institute, Shandong First Medical University and Shandong Academy of Medical Sciences, Jinan, Shandong, China; 3 Department of Radiation Oncology, The Third Affiliated Hospital of Shandong First Medical University, Jinan, Shandong, China

**Keywords:** brain metastases, chemoradiotherapy, PCI, risk factors, small cell lung cancer

Author “Zheng-qiang Yang” was erroneously assigned as corresponding author. The correct corresponding author is “Chao Gao”.

The original version of this article has been updated.

